# Current situation of laboratory medicine in Spain

**DOI:** 10.1515/almed-2022-0013

**Published:** 2022-03-01

**Authors:** Imma Caballé

**Affiliations:** Spanish Society of Laboratory Medicine, Barcelona, Spain; Catlab, Viladecavalls, Spain

**Keywords:** clinical laboratory, professionals, White Paper

Since the outbreak of the COVID-19 pandemic, laboratory medicine has played and a key role and contributed decisively to protect public health. However, this pandemic has also demonstrated the limitations and emerging needs of this field of medicine.

Awareness of the current situation in laboratory medicine is far from what is desirable. The statistical data currently available is partial and limited. Currently, a standard portfolio of services with a standardized nomenclature and evaluation of laboratory activity is not available. For this reason, assessing the volume, cost, performance, organization, quality, and staffing of clinical laboratories is challenging.

The Spanish Society of Laboratory Medicine, cognizant of the situation, launched an initiative to prepare a White Paper [1]. The purpose was to draw a picture of the situation and provide guidance for overcoming the limitations identified. Given that a White Paper provides a description at a given time point, we chose year 2019, before the pandemic, as a reference.

Participants made a considerable effort to make this initiative a success. To complete the questionnaire, participants had to review and update data from their laboratories. Firstly, a list of active laboratories in Spain was compiled, as a database containing reliable, updated data about the laboratories in active service in Spain was not available. The purpose was to guarantee the representativity of the sample in terms of volume of activity and geographical location. The White Paper provides data for 174 clinical laboratories, which received 55.9 millions of test requests and performed 800 millions of determinations in 2019. The determinations performed in the survey of clinical laboratories have an estimated representativity of 90%. Nevertheless, the quality of the information provided will progressively improve as updated versions of the White Paper are made available.

The White Paper also contains crucial information about the situation of laboratory professionals. To date, the number and type for laboratory professionals was unknown, since the Spanish Ministry of Health only provided data about laboratory physicians. This White Paper provides a picture of the situation of laboratory professionals and sends a clear message to decision-makers. Clinical laboratories in Spain need to renew their staff. Notably, 20–25% of laboratory physicians will retire in the next five years (23% are older than 60 years). Over a broader time horizon, more than half the staff of clinical laboratories will retire in 15 years. It is vital that laboratory training programs are updated to be able to meet the expected demand for laboratory professionals. Otherwise, this sector will be negatively affected by a worrying scarcity of laboratory professionals.

Additionally, the White Paper emphasizes the need that the specialties of clinical analysis and clinical biochemistry be merged into a single specialty, where specialized training is provided and the number of vacancies is increased. In Europe, the situation is heterogeneous, as summarized in a recent article [2].

Providing proof of the quality of laboratory results is crucial, as reliable results ensure optimal clinical decision-making. ISO certification is a reference for quality standards. However, only 49% of laboratories have been awarded ISO 9001 certification, and 85 laboratories have ISO 15189 certification. For this reason, SEQC advocates that ISO 15189 certification be made mandatory. This is the most important certification in the clinical laboratory sector, since it guarantees the reliability of results, and it is compulsory in numerous European countries.

Technological innovation is a key to the future of clinical laboratory. The increasing level of automation achieved in laboratories is a hallmark, since it optimizes laboratory performance. Indeed, the participating laboratories have made a considerable effort of digitization and automation of their systems: 90% of centers have achieved a level of automation above 60%. In addition, more than 75% of test requests are electronic in 82% of centers.

Laboratory Medicine is playing a major role in the so-called bio-revolution [3]. Molecular biology and genomics have open a range of sequencing possibilities that make it possible to perform tests that in the past could only be performed in research studies. The application of Big Data and Artificial Intelligence to clinical laboratory will help establish reference values, guarantee the quality of results, support clinical decisions and help monitor public health. These innovations require a new approach to laboratory medicine. This debate has already started in Europe [4].

Finally, clinical laboratories are developing a stronger environmental commitment. According to the data obtained from the questionnaire, an environmental management program is available in around 90% of laboratories, and a waste disposal protocol has been established in 98%. However, laboratory liquid waste disposal is still a pending issue.

This White Paper is the fruit of cooperation between the members of the Spanish Society of Laboratory Medicine. We hope it will contribute to achieve improvements and enhance our capacity to confront immediate and future challenges of laboratory medicine.
